# The Human Polyoma JC Virus Agnoprotein Acts as a Viroporin

**DOI:** 10.1371/journal.ppat.1000801

**Published:** 2010-03-12

**Authors:** Tadaki Suzuki, Yasuko Orba, Yuki Okada, Yuji Sunden, Takashi Kimura, Shinya Tanaka, Kazuo Nagashima, William W. Hall, Hirofumi Sawa

**Affiliations:** 1 Department of Molecular Pathobiology, Hokkaido University Research Center for Zoonosis Control, Sapporo, Japan; 2 Global COE Program for Zoonosis Control, Hokkaido University, Sapporo, Japan; 3 Career-Path Promotion Unit for Young Life Scientists, ICDO, Kyoto University, Kyoto, Japan; 4 Laboratory of Comparative Pathology, Hokkaido University School of Veterinary Medicine, Sapporo, Japan; 5 Laboratory of Cancer Research, Department of Pathology, Hokkaido University School of Medicine, Sapporo, Japan; 6 Centre for Research in Infectious Diseases, University College Dublin, Dublin, Ireland; University of Michigan, United States of America

## Abstract

Virus infections can result in a range of cellular injuries and commonly this involves both the plasma and intracellular membranes, resulting in enhanced permeability. Viroporins are a group of proteins that interact with plasma membranes modifying permeability and can promote the release of viral particles. While these proteins are not essential for virus replication, their activity certainly promotes virus growth. Progressive multifocal leukoencephalopathy (PML) is a fatal demyelinating disease resulting from lytic infection of oligodendrocytes by the polyomavirus JC virus (JCV). The genome of JCV encodes six major proteins including a small auxiliary protein known as agnoprotein. Studies on other polyomavirus agnoproteins have suggested that the protein may contribute to viral propagation at various stages in the replication cycle, including transcription, translation, processing of late viral proteins, assembly of virions, and viral propagation. Previous studies from our and other laboratories have indicated that JCV agnoprotein plays an important, although as yet incompletely understood role in the propagation of JCV. Here, we demonstrate that agnoprotein possesses properties commonly associated with viroporins. Our findings demonstrate that: (i) A deletion mutant of agnoprotein is defective in virion release and viral propagation; (ii) Agnoprotein localizes to the ER early in infection, but is also found at the plasma membrane late in infection; (iii) Agnoprotein is an integral membrane protein and forms homo-oligomers; (iv) Agnoprotein enhances permeability of cells to the translation inhibitor hygromycin B; (v) Agnoprotein induces the influx of extracellular Ca^2+^; (vi) The basic residues at amino acid positions 8 and 9 of agnoprotein key are determinants of the viroporin activity. The viroporin-like properties of agnoprotein result in increased membrane permeability and alterations in intracellular Ca^2+^ homeostasis leading to membrane dysfunction and enhancement of virus release.

## Author Summary

Most non-enveloped viruses exit their host cells following cell lysis, which involves breakdown of the cell membrane and death of the host cell, and which is presumably the final result of an increase in plasma membrane permeability. JC virus (JCV) is the causative agent of progressive multifocal leukoencephalopathy (PML) and belongs to the polyomavirus family, which have non-enveloped virions. The extracellular release of mature progeny polyomavirus virions has been suggested to occur when cells disintegrate or rupture; however, the molecular mechanism(s) employed by JCV to induce cell lysis and facilitate virion release remain elusive. Viroporins are a group of proteins that modify the permeability of cellular membranes and promote the release of viral particles from infected cells. These proteins are not essential for the replication of viruses, but their presence often enhances virus growth. Here, we demonstrate that the JCV agnoprotein forms homo-oligomers as an integral membrane protein and acts as a viroporin, and that expression of agnoprotein results in plasma membrane permeabilization and virion release. These observations suggest that the process of virion release of this non-enveloped DNA virus is highly regulated by a single viral protein.

## Introduction

Replication of viruses involves an extracellular step in the viral life cycle, involving the release of virus particles from infected cells and subsequent infection of target cells. Most non-enveloped viruses exit their host cells by lytic process, which involves breakdown of the cell membrane and is associated with cell death [1].

JC virus (JCV) is the causative agent of progressive multifocal leukoencephalopathy (PML), and belongs to the family of polyomaviruses, which also includes simian virus 40 (SV40) and BK virus (BKV). Polyomaviruses have non-enveloped icosahedral-shaped capsids of about 40 nm in diameter. It has been previously suggested that extracellular exit of the mature progeny virions of SV40 and JCV, which efficiently proliferate in the nuclei, occurs when cells disintegrate or rupture as part of their dying process. However, it remains unclear whether these virions employ specific molecular mechanism(s) which may contribute to or regulate cell lysis. Cell lysis is presumably the ultimate result of an increase in plasma membrane permeability [2]. It has long been considered that either the bulk of viral gene expression or the formation and accumulation of progeny virus particles may be responsible for enhancing membrane permeability and lysis of the cell. However, more recent studies have suggested that individual viral proteins may contribute to the enhancement of plasma membrane permeability and release of progeny virions of a number of non-enveloped viruses, including poliovirus, rotavirus, and coxsackievirus [3] [4] [5] [6] [7]. It has been shown that several viral proteins with membrane permeabilizing properties share common characteristics and these proteins have been named “viroporins” [6]. The proteins share a number of features in structure and function. Viroporins are integral membrane proteins which vary in size from about 60–120 amino acids, possessing at least one hydrophobic stretch able to form an amphipatic α-helix. Viroporins interact with membranes to increase permeability to ions and other small molecules [2] [6]. After their insertion into membranes, viroporins tend to oligomerize to create a hydrophilic pore [8] [9] [10] [11], and these activities result in release of progeny virions.

The late coding region of JCV encodes a small and basic regulatory protein, agnoprotein, whose functions in the virus life cycle remains unclear. Studies on the SV40 agnoprotein have suggested that the protein may contribute to viral replication at various stages including transcription, translation, and processing of late viral proteins [12] [13] [14] [15], assembly of virions [16] [17] [14], and viral propagation [18] [19]. Agnoprotein has been shown to localize to the cytoplasmic and perinuclear regions in infected cells [20], whereas the other viral proteins are exclusively expressed in the nuclei [21] [20]. Recent observations also suggest that JCV agnoprotein may be involved in release of progeny virions and promote the propagation of JCV [22] [23] [24]. Furthermore, a small interfering RNA (siRNA) specific for agnoprotein mRNA was found to inhibit JCV infection [25] [26].

In the present study, we have investigated the role of the JCV agnoprotein in virion release. Comprising some 71 amino acids, agnoprotein has a hydrophobic transmembrane domain that interacts with and expands the lipid bilayer as homo-oligomers. Furthermore, we have found that agnoprotein acts as a viroporin and its expression induced plasma membrane permeabilization and promoted JCV progeny virion release.

## Results

### An Agnoprotein Deletion Mutant Has a Defect in Virion Release and Viral Propagation

**Figure 1:**
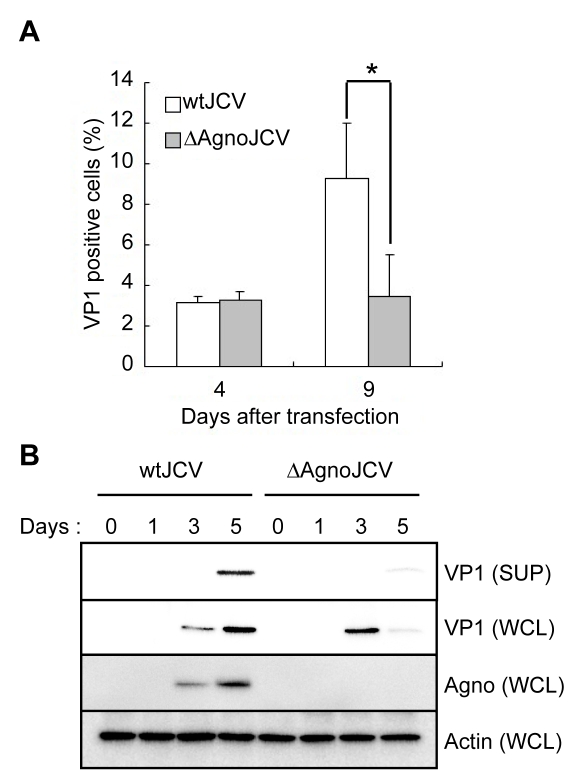
Agnoprotein facilitates virion release and enhances viral propagation. (A) The agnoprotein deletion mutant virus (ΔAgnoJCV) fails to promote viral propagation. Viral growth was monitored by indirect immunofluorescence of VP1. Results shown in the graph were created by determining the average proportion of VP1-positive cells in 3 to 10 microscopy fields counted. Significance of changes were analyzed by student's *t*-test and indicated by an asterisk (*p<0.001). (B) Agnoprotein deletion mutant virus (ΔAgnoJCV) fails to release the virions. Whole cell lysates (WCL) and culture supernatants (SUP) from SVG-A cells transfected with wtJCV or ΔAgnoJCV at the indicated days post-transfection were analyzed by immunoblotting with anti-VP1, anti-agnoprotein and anti-actin antibodies. Culture supernatants were concentrated to 25 times by centrifuging. Agnoprotein expression was confirmed by immunoblotting.

Agnoprotein plays critical roles in viral propagation at multiple steps in JC virus replication [27] [22] [23] [24]. Therefore, we compared the propagation properties of an agnoprotein deletion mutant (ΔAgno) virus with those of the wild type (WT) virus by using a JCV growth assay, which is an established method for measurement of JCV infectivity [28] [24]. In the ΔAgno mutant, the translation initiation codon (ATG) was replaced with the stop codon (TAA), and the mutant does not produce agnoprotein. VP1 protein of cells transfected with ΔAgno mutant was localized in the nuclei similar to that of WT Agno transfected cells (Figure S1) at 4 days after transfection, suggesting that agnoprotein did not affect the intracellular localization of VP1 at this time point. Although the percentage of VP1 of JCV positive cells transfected with either WT or ΔAgno mutant was similar at 4 days after transfection, the percentage of VP1 positive cells transfected with ΔAgno mutant was significantly lower than that of WT at 9 days after transfection (Figure 1A), suggesting that the viral propagation was inhibited in the absence of agnoprotein and which is in accordance with previous reports [27] [25] [24]. We also found that the expression level of VP1 in the culture supernatant from the cells transfected with ΔAgno mutant was substantially decreased compared to that of WT (SUP in Figure 1B). Together, these observations show that agnoprotein plays an important role in the release of virions from infected cells.

### Agnoprotein Localizes at the ER

**Figure 2:**
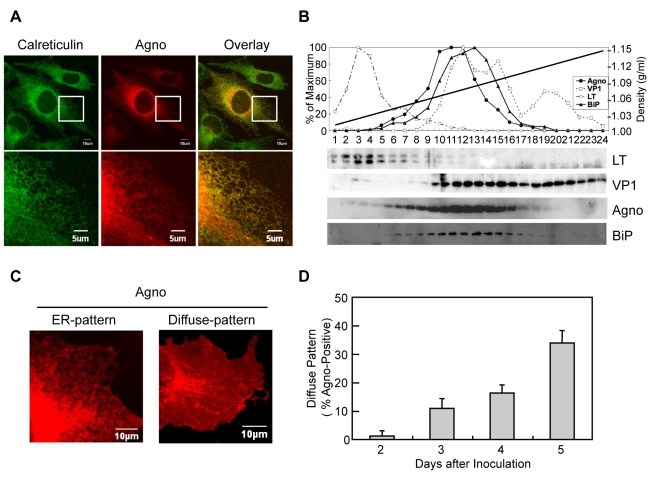
Agnoprotein localized at the ER. (A) Agnoprotein localizes in the ER. Confocal microscopy showed the colocalization of agnoprotein (Agno) and Calreticulin (ER marker) in JCV infected SVG-A cells. The boxed areas in the upper panel are shown at higher magnification in the lower panel. (B) The dominant co-localization of agnoprotein and BiP (ER marker) in JCV infected cells was shown by iodexanol density gradient analysis. (C and D) The intracellular localization of agnoprotein was analyzed at various times after infection by confocal microscopy. Almost all agnoprotein distribute as the ER pattern (C left panel), and there were a few cells with the diffuse pattern (C right panel) at the early stages of infection. The percentage of cells with the diffuse pattern was found to increase in a time-dependent manner (D). Scale bars, 10 µm.

Previously, we reported that agnoprotein is mainly localized in the cytoplasm and perinuclear regions of JCV infected cells [27] [20] [23]. To delineate in more detail the intracellular localization of agnoprotein, we performed immunofluorescence studies using human fetal glial SVG-A cells infected with JCV at 3 days after infection. Confocal microscopy revealed that agnoprotien immunoreactivity was present in the perinuclear region and extended into the cytoplasm in a mesh-like pattern (Figure 2A and S1). Although its localization was relatively heterogenous, most of the agnoprotein consistently overlapped with that of calreticulin, an ER marker and specifically in the peripheral regions of the cytoplasm (Figure 2A). We also performed iodixanol density gradient analysis of the membrane fraction of JCV-infected IMR-32 cells. In contrast to other JCV encoded proteins, such as VP1 and Large T antigen, agnoprotein and BiP, which is an ER marker, were detected in the same fractions (Figure 2B), suggesting that agnoprotein predominantly localizes at the ER. We next examined the intracellular-localization of agnoprotein at various time after infection. It could be shown that almost all agnoprotein was distributed at the ER at 2 days after infection, which is designated as an ER pattern (Figures 2A and C). However, at the later stages (at 5 days after infection) of infection the localization of agnoprotein in the ER changed to become more diffusely localized in the cytoplasm (Figures 2C and D) (designated as diffuse pattern). These observations suggested that while agnoprotein originally localizes at the ER, it's intracellular localization changes to involve other intracellular compartments in time-dependent manner.

### Agnoprotein is an Integral Membrane Protein and Localizes at Plasma Membrane

**Figure 3:**
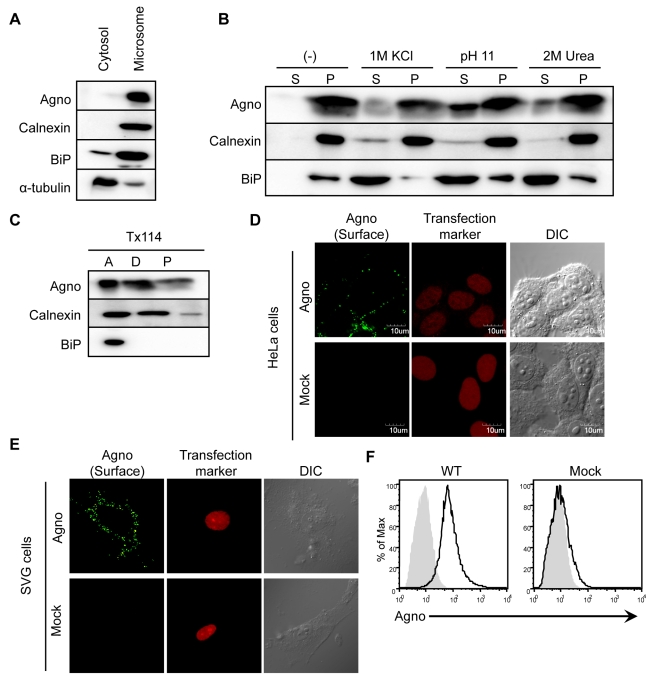
Agnoprotein also localizes at the plasma membranes as an integral membrane protein. (A) Homogenates from 293AG cells which are agnoprotein-inducible cell lines with doxycycline (DOX) treatment were subjected to subcellular fractionation as described in the Materials and Methods. The cytosolic and microsomal fractions were analyzed by immunoblot analysis using antibodies to agnoprotein, calnexin (ER marker), BiP (ER marker), and α-tubulin (cytosolic marker). (B) Microsomes were incubated for 30 min on ice with homogenization buffer alone (−), or with buffer containing 1 M KCl, or 2 M urea. The microsomes were also treated with buffer containing 0.2 M sodium carbonate (pH 11.0). Following treatment, the microsomes were separated into supernatant (S) and pellet (P) fractions by ultracentrifugation. (C) Microsomes were also subjected to Triton X-114 phase separation, and aqueous phase (A, lane 1), detergent phase (D, lane 2), and insoluble aggregates (P, lane 3) were obtained. (D and E) HeLa (D) and SVG-A (E) cells were transfected with pERedNLS-Agno or control vectors. Cell surface agnoprotein on living cells was detected by incubation with antibody against the agnoprotein C-terminal region, followed by staining with Alexa 488-labeled anti-rabbit IgG antibody. DsRed signals in the nuclei represent a marker of transfection. (F) Flow cytometry analysis of cell surface expression of agnoprotein in 293T cells transfected with pCXSN-Agno (WT, left panel) or vector control (Mock, right panel). The black solid line represents anti-agnoprotein antibody staining and the light gray shade represents control rabbit IgG staining, followed by staining with FITC-labeled anti-rabbit IgG antibody.

Next, we attempted to define in more detail the properties of agnoprotein. Analysis of subcellular fractions of 293AG cells which are agnoprotein-inducible cell lines with doxycycline (DOX) treatment [22] showed that agnoprotein was mainly detected in the microsomal compared with the cytosol fractions (Figure 3A). We then examined the interaction between agnoprotein and microsomes by treatment of the microsome membrane fractions with various chemical reagents that selectively disrupt interactions on the surface without extracting proteins from phospholipids bilayers (Figure 3B). Treatments with high salt concentration (1 M KCl) and sodium carbonate buffer (pH 11), as well as incubation with 2 M urea, all abolished the membranous association of BiP which has no hydrophobic transmembrane domain (Figure 3B, lower panel). In contrast, agnoprotein and calnexin, which have a hydrophobic transmembrane domain, were not removed from the microsome membrane even in the presence of these reagents (Figure 3B, upper and middle panels) suggesting that agnoprotein is embedded within the lipid bilayer. Consistent with this, agnoprotein was recovered in the detergent phase along with calnexin in the phase separation experiments using Triton X-114 [29] [30] (Figure 3C). As previously shown the intracellular localization of agnoprotein changes in time-dependent manner (Figures 2C and D). The anterograde transit of vesicular proteins within the early secretory pathway is known to connect the ER to plasma membrane trafficking [31]. To determine if agnoprotein is involved in the secretory pathway, we examined the steady-state cell surface expression of agnoprotein. Non-permeabilized HeLa cells transfected with WTAgno were subjected to immunofluorescence staining with antibody which recognizes the agnoprotein C-terminus [20]. Agnoprotein was detected at the plasma membranes of both non-permeabilized HeLa (Figure 3D) and SVG-A (Figure 3E) cells. The steady-state cell surface expression of agnoprotein was further supported by flow cytometry studies (Figure 3F). These observations suggested that agnoprotein is in part co-translationally inserted into the ER membrane and transported to the plasma membrane at the later stage of infection with the C-terminus of agnoprotein at the extracellular surface.

### The N-terminal Region of Agnoprotein is Necessary for Targeting the ER

**Figure 4:**
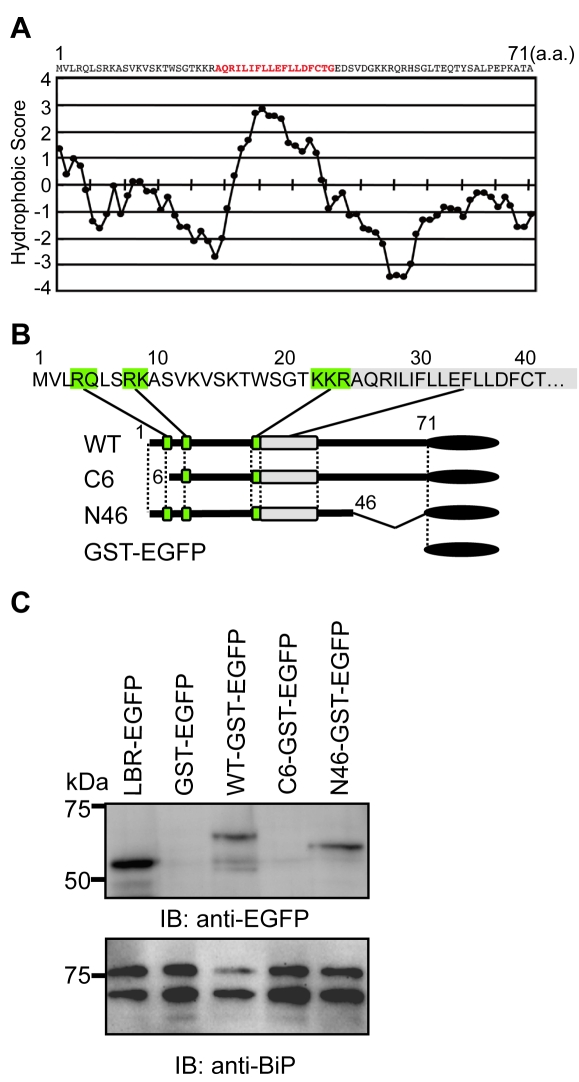
The N-terminus of agnoprotein is necessary for targeting to the ER. (A) Hydrophobicity plot of JCV agnoprotein. The plot was drawn using the Lyte-Doolittle method of calculating hydrophilicity over a window length of seven [54]. (B) The N-terminal region of agnoprotein is characterized by the presence of positively charged residues. Schematic representation of GST-EGFP fusion constructs of wild type (WT) agnoprotein and its various mutants. The green boxes indicate the basic amino acid clusters, which could be important for determining the orientation to the membrane. A gray box indicates a hydrophobic amino acid stretch. (C) Immunoblot analyses of GST-EGFP–fused agnoprotein and its mutants prepared from the ER-nuclear fraction of transfected HEK293 cells prepared by sucrose density centrifugation as described in the Materials and Methods. WT and N46 mutant were detected in the ER-rich fractions, whereas the C6 mutant and GST-EGFP were not.

We next sought to identify the motif(s) of agnoprotein which are necessary for targeting the ER. A Kyte and Doolittle hydrophobicity plot of agnoprotein revealed that the middle region of agnoprotein contains the only hydrophobic region. This is comprised of 18 amino acids and would serve as a potential transmembrane segment (Figure 4A). To determine the domain of agnoprotein important for its ER distribution, we generated a series of mutants as fusion proteins with the GST-EGFP tag (∼50 kDa) (Figure 4B). Immunoblot analyses of GST-EGFP–fused agnoprotein and its mutants were prepared from the ER-nuclear fraction of transfected HEK293 cells by sucrose density centrifugation. WT and N46 mutant were detected in the ER-rich fractions, but the C6 mutant and GST-EGFP were not (Figure 4C). This result suggested that the N-terminus of agnoprotein was necessary for targeting to the ER.

### Both the Basic Amino Acid Cluster and Hydrophobic Regions of Agnoprotein are Required for ER Targeting

**Figure 5:**
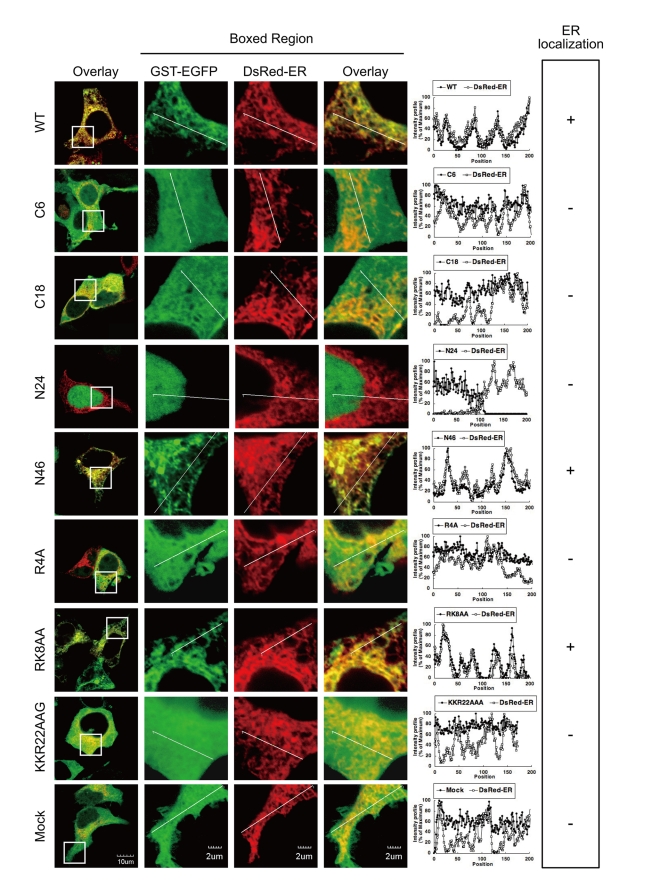
The basic amino acids of the N-terminus and hydrophobic region are necessary for the ER targeting. WT, N46, and RK8AA mutants localized at the ER. Subcellular localization in 293T cells transiently transfected with the constructs described in Figure S3 and pDsRed-ER were analyzed by confocal microscopy. The boxed areas in the left panel are shown at higher magnification in the right panel. Graphs are relative fluorescent intensities along the white lines of higher magnification images. The fluorescent intensities of GST-GFP fused with WT, N46, and RK8AA mutants corresponded with those of DsRed-ER, indicating that WT, N46, and RK8AA mutants localized at the ER. In contrast, the fluorescence intensities of GST-GFP fused with the other mutants were not in accordance with those of DsRed-ER.

We have demonstrated that agnoprotein has an extracellular carboxyl terminal (C-terminal) domain (Figures 3D, E, and F) and that the N-terminal region of agnoprotein was important for the ER targeting (Figure 4C). These results suggest that agnoprotein might have a cytoplasmic domain at the amino terminus and should be considered as an integral transmembrane protein type II [32]. In this situation positively charged amino acids at the N-terminal region would be important for determining the orientation of the transmembrane segment [33]. Agnoprotein has some basic residues in the N-terminal 24 amino acids, and to determine the residues responsible for the subcellular localization of agnoprotein, amino acid point mutations of agnoprotein where generated by site-directed mutagenesis (Figure S3), and the subcellular localizations in 293T cells transiently transfected with these constructs and pDsRed-ER were analyzed by confocal microscopy (Figure 5). The fluorescent intensities of GST-GFP fused with WT and the N46, and RK8AA mutants corresponded with those of DsRed-ER, indicating that WT, N46, and RK8AA mutants localized at the ER. In contrast, the fluorescence intensities of GST-GFP fused with the other mutants did not correlate with those of DsRed-ER. These observations demonstrated that the basic amino acid residues of the N-terminal region of agnoprotein are necessary for its subcellular localization and that agnoprotein is indeed a type II integral transmembrane protein.

### Agnoprotein Forms Homo-Oligomers

**Figure 6:**
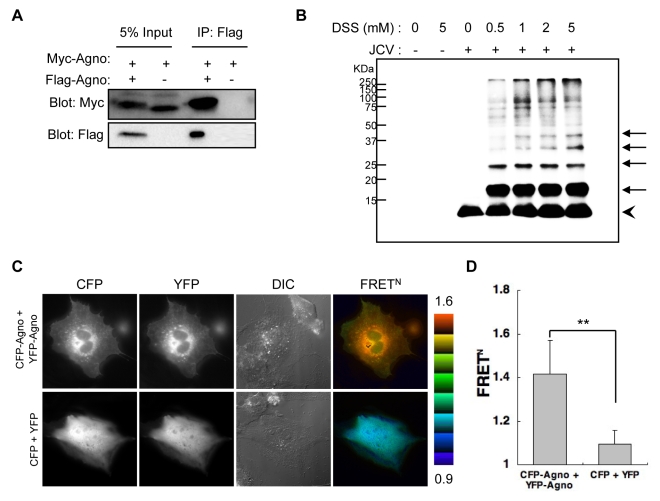
Agnoprotein forms homo-oligomers. (A) 293T cells were transfected with Myc-agnoprotein/Flag-agnoprotein or Myc-agnoprotein alone. Cell lysates were subjected to immunoprecipitation with antibodies to Flag (IP: Flag). (B) SVG-A cells uninfected or infected with JCV [JCV (−) or JCV (+), respectively] were reacted with DSS cross-linker at the indicated concentration (0, 0.5, 1, 2, or 5 mM). Triton X-100 soluble extracts were resolved by SDS-PAGE, and monomer (arrowhead) and oligomers (arrows) of agnoprotein were detected by immunoblot analysis using anti-agnoprotein antibody. (C and D) Intermolecular FRET in SVG-A cells expressing agnoprotein fused with CFP (CFP-Agno) or YFP (YFP-Agno). Pseudocolour images represent normalized values (FRET^N^), with the intensity of each color indicating the mean intensity of FRET and the total spillover from the relevant FRET partners as indicated at the bottom of the photographs. The upper (1.6) and lower (0.9) limits of the ratio range are shown at the right. Numbers of samples: CFP + YFP, n = 53; CFP-Agno + YFP-Agno, n = 59 (**p<0.0001).

Viroporins are responsible for membrane leakiness late in infection. Typically, viroporins are comprised of some 60–120 amino acids, contain a highly hydrophobic domain, and may also contain a stretch of basic amino acids. The insertion of viroporins into membranes is followed by their oligomerization and this is thought to be critical for membrane destabilization [2] [6]. Agnoprotein is an integral membrane protein that has some of the expected features of viroporins. To determine whether agnoprotein acts as a viroporin, we examined the formation of agnoprotein homo-oligomers by several methods: 1) coimmunoprecipitation of epitope-tagged agnoproteins (Figure 6A), 2) chemical cross-linking of SVG-A cells infected with JCV (Figure 6B), and 3) intermolecular fluorescence resonance energy transfer (FRET) studies using agnoprotein fused with either Venus (YFP-Agno) or sECFP (CFP-Agno) at the NH2-terminus (Figures 6C and D). Myc-tagged agnoprotein was coimmunoprecipitated with Flag-tagged agnoprotein using anti-Flag antibody (Figure 6A), suggesting that agnoprotein forms homo-oligomers. SVG-A cells infected with JCV were incubated with a crosslinker reagent, disuccinimidyl suberate (DSS) or buffer alone, and homogenized with Triton X-100 containing lysis buffer following immunoblotting. The immunoblotting revealed that there were multiple bands, which migrated with the expected positions of monomers (approximately, 8 kDa), dimers (16 kDa), trimers (24 kDa), tetramers (32 kDa), and pentamers (40 kDa) (Figure 6B). Furthermore, FRET assays confirmed that agnoprotein forms homo-oligomers at the cytoplasmic organelles where agnoprotein is known to localize (Figures 6C and D).

### Expression of Agnoprotein Results in Enhanced Plasma Membrane Permeability

**Figure 7:**
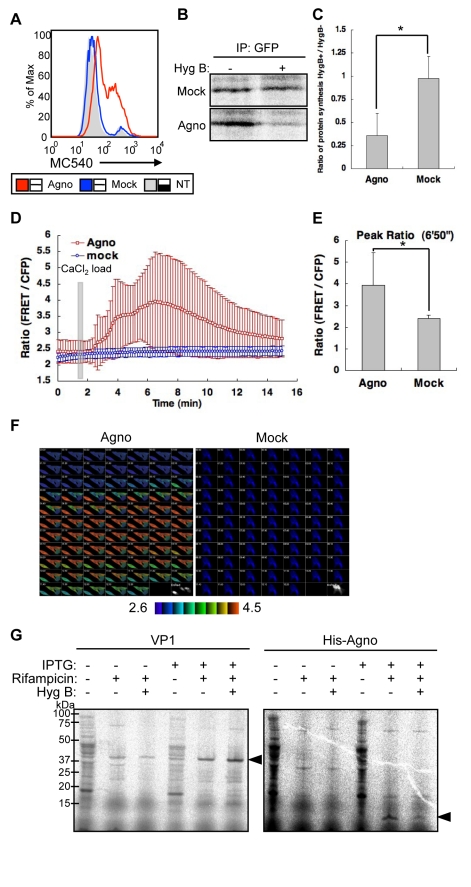
Agnoprotein impairs membrane integrity. (A) HeLa cells were transfected with pCXSN-Agno (Agno) or pCXSN (Mock) and assayed by flow cytometry for MC540 binding. NT: not transfected. (B) HeLa cells were either transfected with control vector (Mock) or pCFPNLS-Agno (Agno) for 72 h. The cells were pretreated with or without HygB (400 µg/ml), radiolabeled with [^35^S] Met-Cys, and incubated in the absence (+) or presence (−) of HygB for 2 h. The cell extracts were harvested, and the marker protein was immunoprecipitated with an anti-GFP antibody and analyzed by SDS-PAGE. (C) The results of (B) were quantified by Image Gauge V3.2 software. The bar graph represents the protein synthesis ratio in the presence to the absence of HygB, and corresponds to an average of triplicates ± S.D. Significance was analyzed by Student's *t*-test and is indicated by asterisk (*p<0.01). (D) Intracellular Ca^2+^ concentration was measured by an intramolecular FRET-based Ca^2+^ indicator. HeLa cells were transfected with FRET probe (YC3.60) and coexpressed pERedNLS-Agno (Agno) or pERedNLS (Mock). Permeability to Ca^2+^ in agnoprotein (Agno)- or mock (Mock)-expressed cells was evaluated by the change in FRET ratio induced by the addition of 5 mM CaCl_2_ to the extracellular medium at 100 sec after the start of recording. Results of cells with agnoprotein or mock are the mean ±SD from 28 or 39 cells, respectively. (E) The bar graph represents the highest FRET ratio at 6′50″ (*p<0.001). (F) Ratio images of FRET/CFP were created to represent FRET efficiency. The upper (4.5) and lower (2.6) limits of the ratio range are shown at the bottom. (G) Agnoprotein permeabilizes bacterial cells. E. coli cultures were pulsed for 10 min with [35S]Met-Cys in the absence or presence of hygromycin B with or without inducing the expression of VP1 (left panel) or Agno with a NH2-terminal His tags (His-Agno, right panel) with IPTG. Rifampicin was included during the induction to inhibit endogenous E. coli protein synthesis. Bacteria were sedimented, lysed, and resolved by 4–12% reducing SDS-PAGE followed by autoradiography.

To determine whether agnoprotein is capable of producing permeabilization of the plasma membrane, we examined plasma membrane structures using Merocyanine 540 (MC540), which is a lipophilic fluorescence dye that binds to the outer leaflet of plasma membranes [34]. Several studies in model membranes have demonstrated that its fluorescence characteristics are sensitive to subtle differences in lipid packing [35] [36]. We used flow cytometry to examine MC540 binding of HeLa cells transfected with agnoprotein (Agno) or mock plasmid (Mock), or without transfection (NT). The MC540 intensity in Agno cells was significantly higher than in Mock or NT cells (Figure 7A), suggesting that agnoprotein disrupts lipid packing and modifies the structure of the plasma membrane. To extend these findings, we performed a Hygromycin B (HygB) permeability assay. HygB is a general inhibitor of translation, and intact mammalian cells are impermeable to HygB when exposed for short time periods at low concentrations. However, HygB will enter mammalian cells with permeabilized plasma membranes [37]. HeLa cells were transfected with pCFPNLS-Agno or mock plasmid. The plasmid contains the internal ribosome entry site (IRES) of the encephalomyocarditis virus (ECMV) between the multiple cloning sites (MCS) and the CFPNLS [sECFP with nuclear localization signal (NLS) of simian virus 40 large T-antigen fused to its C-terminus] following the immediate early cytomegalovirus promoter which permits both the inserted gene in the MCS and the CFPNLS gene to be translated from a single bicistronic mRNA. The cells transfected with the plasmids were identified by expression of sECFP in the nucleus. Following incubation for 72 h, cells were then radiolabeled with [^35^S] Met-Cys for 2 h in the absence or presence of HygB. Cell extracts were harvested, and the protein synthesis marker CFPNLS was detected by immunoprecipitation with an anti-GFP antibody that cross-reacts with CFP. In cells transfected with pCFPNLS-Agno, radio-labeled CFPNLS levels were markedly decreased in the presence of HygB; in contrast in mock transfected cells, CFPNLS levels were unaffected by HygB (Figures 7B and C). Thus, agnoprotein expression clearly altered the cell membrane permeability for HygB. This increase in membrane permeability for HygB was also confirmed using SVG-A cells (Figure S4A and S6A).

Viral proteins, which can enhance plasma membrane permeabilization to small molecules such as HygB, can also induce an increased permeability to ions, such as Ca^2+^ [2] [6]. We measured the influx of extracellular calcium using the intramolecular FRET-based Ca^2+^ indicator, yellow cameleon 3.60 (YC3.60) [38]. Ca^2+^ influx into cells was induced by addition of CaCl_2_ to the extracellular medium in the presence, but not in the absence, of agnoprotein (Figures 7D, E, and F; the video files can be viewed as supplementary Videos S1 and S2). These studies show an increased permeability for Ca^2+^ and it can be concluded that agnoprotein enhances membrane permeability to ions as well as to small molecules.

We also investigated whether agnoprotein possesses inherent properties that result in cell permeabilization and death independent of the signaling pathways within the host organism. To address this question, the cell permeability of Escherichia coli (E. coli) was examined after expression of either agnoprotein containing His tag at the NH2-terminus or VP1 without a His tag driven by a T7 promoter as has been previously described for SV40 VP4 [39]. The integrity of the E. coli double-membrane barrier was analyzed by determining the sensitivity of protein synthesis to HygB. To inhibit the E. coli RNA polymerase and prevent the synthesis of endogenous bacterial proteins, rifampicin was also added during the induction of E. coli growth. The expression of VP1 had no effect on E. coli membrane permeability, as the synthesis of ^35^S-labeled VP1 was not inhibited by addition of HygB (Figure 7G, left panel, arrow head). In contrast, agnoprotein expression resulted in the permeabilization of E. coli, as the synthesis of ^35^S-labeled agnoprotein was clearly inhibited in the presence of HygB (Figure 7G, right panel, arrow head). Cellular membrane permeabilization can lead to cell lysis or cell death. Therefore, we investigated the viability of E. coli after the induction of either VP1 or agnoprotein expression to determine whether these viral proteins possess lytic properties. The induction of VP1 expression did not cause obvious lysis of the bacteria, as no decrease in the OD was observed (Figure S4B) Instead, the induction of agnoprotein showed a relatively low increase rate of OD compared to that of VP1 (Figure S4B). These observations indicated that agnoprotein expression does not cause lysis but would appear to have some toxic effect on E. coli growth. While the molecular basis of this effect is unclear it may be due to or be related to the permeabilization of the bacterial membrane.

### Arg-8 and Lys-9 are Necessary for the Viroporin Activity of Agnoprotein

**Figure 8:**
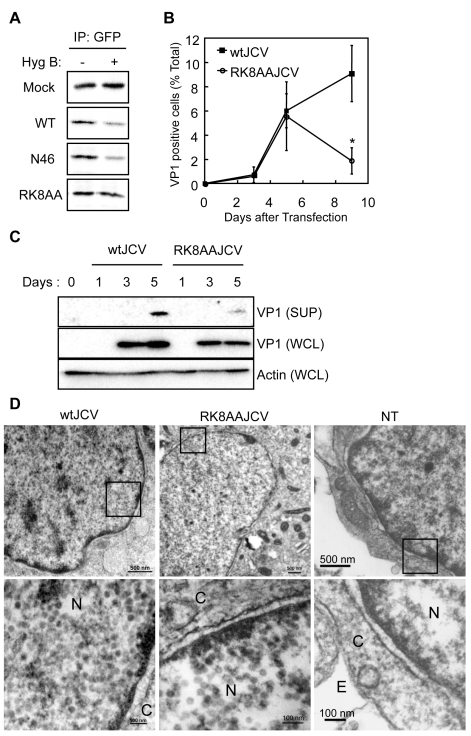
“RK” residues of Agnoprotein are necessary for viroporin activity. (A) HygB permeability assays with HeLa cells transfected with control vector (Mock), pCFPNLS-Agno (Agno), pCFPNLS-N46 (N46), or pCFPNLS-RK8AA (RK8AA). (B) Viral growth was monitored by indirect immunofluorescence of VP1 (*p<0.001). (C) Whole cell lysates (WCL) and culture supernatants (SUP) from SVG-A cells transfected with wtJCV or RK8AAJCV at the indicated days post-transfection were analyzed by immunoblotting with an anti-VP1 antibody. Culture supernatants were concentrated to 25 times by centrifugation. (D) Electron microscopic analysis of wtJCV- or RK8AAJCV–transfected SVG-A cells. NT: not transfected. The boxed areas in the upper panel are shown at higher magnification in the lower panel. Viral particles of about 40-nm in diameter were in the nuclei (N) of cells transfected with either wtJCV or RK8AAJCV. Scale bars in upper and lower panels, 500 and 100 nm, respectively. C: cytoplasm. E: extracellular space.

To further investigate the viroporin activity, we performed a HygB permeability assay with agnoprotein mutants (N46 mutant and RK8AA mutant), which localize at the ER similar to WT protein (Figure 5). In N46 mutant- or WT Agno-transfected HeLa cells, the amount of radio-labeled CFPNLS was markedly decreased in the presence of HygB compared to its absence (Figure 8A). In contrast, the levels of CFPNLS were unaltered in mock or RK8AA- transfected cells (Figure 8A), suggesting that WT Agno and N46, but not RK8AA, enhances membrane permeabilization. To further demonstrate the importance of this viroporin activity, we generated viral genomes containing the RK8AA mutation of agnoprotein (RK8AAJCV) and performed JCV growth assays. Viral growth was measured by monitoring the percentage of VP1 positive cells (Figure 8B). At 3 and 5 days post-transfection, the percentage of VP1 positive cells were similar in wtJCV- and RK8AAJCV-transfected cells (Figure 8B). However, by 9 days the percentage of VP1 positive cells of RK8AAJCV-transfected cells had significantly decreased compared to that at day 5, whereas that continued to increase in wtJCV-transfected cells (Figure 8B), suggesting that RK8AA mutation of agnoprotein was associated with impaired viral propagation. The amount of VP1 in the culture supernatant of RK8AAJCV-transfected cells was, as expected, substantially decreased (SUP in Figure 8C and S6B). The intracellular localization and expression level of agnoprotein of RK8AAJCV-transfected cells was similar to those of wtJCV-transfected cells (Figures S5A and B). These results suggested that RK8AAJCV has a defect in the release of progeny virus. Transmission electron microscopy was carried out to evaluate whether the RK8AA mutant of agnoprotein influences the virion assembly. This revealed the presence of virus particles of 40-nm in diameter in the nuclei of RK8AAJCV-transfected cells similar to that observed in wtJCV-transfected cells at 5 days post-transfection (Figure 8D, left and middle columns). In contrast, particles were not observed in cells without transfection (Figure 8D, right columns). The virions extracted from the cells transfected with RK8AAJCV were able to infect to the SVG-A cells similar to that of wtJCV and thus were not defective (Figure S5C). These findings show that the inability of the RK8AAJCV to propagate viral infection is due to a defect(s) in virion release. Taken together, Arg-8 and Lys-9 in the NH2-terminus of agnoprotein are necessary for viroporin activities, which permeabilize the cell membrane and release virions from the JCV infected cells.

## Discussion

In this study, we demonstrate that JCV agnoprotein, which primarily localizes at the ER, is also present in the plasma membranes, and that the basic amino acid clusters in the N-terminus are necessary for its localization. In addition, we have demonstrated that agnoprotein acts as a viroporin in that it forms homo-oligomers as an integral membrane protein and that expression of agnoprotein results in plasma membrane permeabilization, which in turn facilitates release of progeny virions. Moreover we show that Arg-8 and Lys-9 in the NH2-terminus of agnoprotein are necessary for the viroporin activity.

### Agnoprotein is an Integral Membrane Protein, which is Primarily Distributed to the ER but also Localizes at the Plasma Membrane

The amino acid sequence of JCV agnoprotein shares ∼60% identity with that of the other polyomaviruses SV40 and BKV. In contrast the sequences of other viral proteins, such as VP1 or large T Ag, are highly conserved (80–90% identity). The C-terminal amino acid sequences of the agnoproteins are unique in the individual viruses, whereas those at the N-terminal 40 amino acids exhibit around 90% identity. As the subcellular localizations of the agnoproteins are almost identical, this suggests the existence of a functional domain in the N-termini. JCV agnoprotein is the only viral transcript expressed in cytoplasm rather than the nucleus, and especially in perinuclear region [20]. In contrast to the other viral late proteins, agnoprotein is not incorporated into in the viral capsid and is undetectable in purified infectious virions.

In this report we demonstrate that JCV agnoprotein is an integral transmembrane protein type II targeted to the organelles in an exocytic route, involving the ER and subsequently localizing to the plasma membrane in time-dependent manner. The integral transmembrane protein type II agnoprotein has a cytoplasmic amino terminus and an extracellular carboxyl terminus [32]. Positively charged amino acids at the N-terminal region are important for determining the orientation of the transmembrane segment [33]. In the N-terminus of agnoprotein, there are several positively charged amino acids, and these residues with the exception of Arg-8 and Lys-9 contribute to the subcellular localization of the protein.

### Agnoprotein Forms Homo-Oligomers and Increases Membrane Permeabilization

Viruses produce a number of injuries during infection of susceptible cells. Some of these affect cell membranes, and a typical feature observed during the replication of a number of animal viruses is enhanced membrane permeability. Several viral gene products are considered to be responsible for these changes and these include proteases, glycoproteins, and viroporins [2] [6]. Viroporins are small, highly hydrophobic, virus-encoded proteins that interact with membranes modifying the cell permeability to ions or other small molecules. Typically, viroporins are comprised of some 60–120 amino acids and contain a highly hydrophobic domain. The insertion of viroporins into membranes followed by their oligomerization leads to membrane destabilization, thus enhancing permeability [2] [6]. We have found that agnoprotein has all of the features of viroporins and is capable of enhancing the membrane permeability for Hygromycin-B and Ca^2+^. In addition, agnoprotein enhanced the plasma membrane binding of MC540 which is a lipophilic fluorescence dye binding to the outer leaflet of plasma membranes [34]. While the changes of binding of MC540 support alterations of membrane structures, the studies do not allow clear definition of any particular mechanism [40]. Our results also show that agnoprotein can permit small molecules [molecular weight (MW) under 1,000 Da] to enter the cytoplasm. Because the MW of virions are much larger than 1,000 Da, they may be released from the cells not through the direct effect of membrane permeabilization but perhaps indirectly by the alteration of cytoplasmic concentrations of monovalent cations, which can provoke membrane depolarization, leading to cell lysis [7]. In SV40, VP2 and VP3, but not VP1, possess membrane-permeabilizing activity in prokaryotic cells [41]. Furthermore, the ability to induce bacterial lysis following permeabilization was an exclusive property of VP3, suggesting that membrane permeability changes induced by VP3, and perhaps VP2 as well, are largely responsible for the necrosis resulting from SV40 infection and that the lytic activity of VP3 may be necessary for the release of progeny virions [41]. In this study we also show that JCV agnoprotein possesses bacterial membrane-permeabilizing activity. However, agnoprotein could not induce bacterial lysis. Thus, is possible that cell-lytic activity may require expression of other viral proteins in addition to agnoprotein during JCV infection.

### Arg-8 and Lys-9 of Agnoprotein Define the Viroporin Activity

Some viroporins contain a stretch of basic amino acids that act as a detergent [6] and agnoprotein has some clusters of basic residues in the NH2-terminal 24 amino acids. The R4A and KKR22AAG mutants of agnoprotein did not distribute to the ER, suggesting that these residues are important for the correct intracellular localization of agnoprotein. Although the RK8AA mutant of agnoprotein localized at the ER as well as WT, this mutant did not have the viroporin activity. These observations indicate that the “RK” residues at amino acid positions 8 and 9 of agnoprotein are not necessary for the intact intracellular localization but contribute to the plasma membrane permeability. These residues may serve as a detergent, modify the membrane structure, or interact with other molecules in host cells. However, the exact functions of these basic amino acids will further investigation and these studies will certainly help elucidate the molecular mechanisms of the viroporin activity.

Furthermore, previous studies with other polyomaviruses have implied a direct interaction of agnoprotein and VP1 [42], and indeed JCV agnoprotein does interact with GST-fused VP1 synthesized in E. coli [our unpublished observation]. These interactions would appear to be in addition to the role of agnoprotein as a viroporin. Thus agnoprotein can be considered to be a multifunctional auxiliary protein [43], and studies on other polyomavirus agnoproteins have suggested that the protein may contribute to a variety of stages of the viral life cycle including assembly of virions and viral propagation [14] [16] [17] [18] [19]. Thus, the function of agnoprotein and the interaction with VP1 will require further investigation to clarify the roles of JCV agnoprotein in virion assembly.

In summary, our studies demonstrate that agnoprotein acts as a viroporin resulting in plasma membrane permeabilization and virion release. The permeabilization induced by agnoprotein is crucial for viral propagation and could be a potential target for a therapeutic intervention in PML. Furthermore, our study highlights that the mechanism of virion release of a non-enveloped DNA virus is highly regulated by a single viral protein.

## Materials and Methods

### Construction of Plasmids

For expression of JCV agnoprotein in mammalian cells, the cDNA of JCV agnoprotein was amplified by polymerase chain reaction (PCR) using a plasmid encoding the JCV genome, pJC1->4pJCV [VG015, Health Science Research Resources Bank (HSRRB)] and subcloned into a pGST-EGFP plasmid including GST into the Kpn I and Bam HI sites of pEGFP-N1 (Clontech Mountain View, CA); pCXSN plasmid which was constructed by removing myc-tag from pCMV-myc (Clontech) and adding Xho I, Sal I, and Not I recognition sites; pCXSN-FlagN which was constructed by adding Flag-tag to pCXSN plasmid at the 5′ region of Xho I site; pCXSN-MycN which was constructed by adding Myc-tag to pCXSN plasmid at the 5′ region of Xho I site; pERedNLS (kindly provided by Dr. M. Matsuda); and pCFPNLS which was constructed by replacing DsRedExpress with sECFP (kindly provided by Dr. A. Miyawaki); pCXSN-YFP/CFP which constructed by insertion of Venus (kindly provided by Dr. A. Miyawaki) or sECFP to the Xho I site of pCXSN and adding linker sequences (RSTGNSADGGGGSGGSGGSGGGSTQGGSSGTGTAAENSGNSRTK) at the 3′ region of Venus or sECFP. Deletion mutants of agnoprotein were constructed by PCR with KODplus polymerase (Toyobo, Tokyo, Japan). The substitution mutants of agnoprotein were constructed by using the QuickChange site-directed mutagenesis kit (Stratagene) according to the manufacture's procedure. The primers used for generating mutants of agnoprotein were (5′ -> 3′): 1, ctcagatctaatggttcttGCCcagctgtcacg and ctggtaccgtagcttttggttcaggcaaagc (R4A); 2, ctcagatctaatggttcttcgccagctgtcaGCTGCTgct and ctggtaccgtagcttttggttcaggcaaagc (RK8AA); 3, gtaaaacctggagtggaactGCAGCAGGAgctcaaaggatttta and taaaatcctttgagcTCCTGCTGCagttccactccaggttttac (KKR22AAG). The following plasmids containing wild type (WT) agnoprotein were used as PCR templates: pGST-EGFP-Agno, pCXSN-Agno, and pERedNLS-Agno. The plasmid containing the genome of JCV Mad1-SVEΔ (pUC19-Mad1SVEΔ) was kindly provided by Dr. W. J. Atwood [28] [44]. Agnoprotein with mutated viral genomes (ΔAgno and RK8AA) were generated by site-directed mutagenesis using PCR with pUC19-Mad1SVEΔ, which has WT agnoprotein, and KODplus polymerase. In the ΔAgno mutant, the translation initiation codon (ATG) of agnoprotein was changed to the stop codon (TAA) by base substitution, and the ΔAgno mutant viral DNA is defective in the production of agnoprotein. Primers used for mutagenesis were (5′-> 3′): 1, TAAgttcttcgccagctgtcacg and ggccagcggtacctgtggaat (ΔAgno). 2, GCTgctttctgtgaaagttagtaaaacctgg and AGCtgacagctggcgaagaaccatg (RK8AA). Mismatched nucleotides are shown in uppercase letters. Successful mutagenesis was confirmed by sequencing. A DsRed-ER expressing plasmid (pDsRed-ER) was obtained from Clontech. All the constructs and materials used in the experiments were described in Table S1.

### Cell Culture and Virus preparation

Human embryonic kidney 293 cells with SV40 T antigen (HEK293T), human cervical carcinoma cells (HeLa), and human neuroblastoma cells IMR-32 cells were obtained from the HSRRB. A JCV-producing cell line (JCI cells [45]) and SV40-transformed human glial SVG-A cells (kindly provided by Dr. W. J. Atwood) [46] were also used. All cells were maintained under 5% CO_2_ at 37°C condition in Dulbecco's minimal essential medium (DMEM) supplemented with 10% heat-inactivated fetal bovine serum (FBS), 2 mM L-glutamine, penicillin, and streptomycin (Sigma). Establishment and maintenance of HEK293 cells expressing JCV agnoprotein in an inducible manner (293AG cells) was described previously [22] [23].

For virus preparation, JCI cells or JC virus-infected SVG-A cells were harvested and suspended in Tris-HCl (pH 7.5) containing 0.2% bovine serum albumin (BSA), frozen and thawed three times, and then treated with 0.05 U/ml of neuraminidase type V (Sigma) at 37°C for 16 h. After incubation at 56°C for 30 min, cell lysates were centrifuged at 1,000×g for 10 min. The supernatant was quantified by hemagglutination (HA) assays and stored at −80°C until use.

### Primary Antibodies

Rabbit anti-JCV agnoprotein, anti-JCV VP1, and anti-JCV Large T polyclonal antibodies were produced as described previously [47] [20] [48]. Alexa 594–labeled anti-agnoprotein antibody was used for double-immunofluorostaining with another rabbit polyclonal antibody. Mouse anti-BiP and anti-Calnexin monoclonal antibodies were purchased from BD Transduction Laboratories. Mouse anti-actin (MAB1501R) monoclonal antibodies were purchased from Chemicon International. Mouse anti-Flag (M2) and anti-alpha tubulin monoclonal antibodies were purchased from Sigma. Goat anti-GFP polyclonal antibody was purchased from Rockland. Rabbit anti-calreticulin polyclonal antibody was purchased from Calbiochem.

### JCV Growth Assay

Mutant and WT viral DNA were linearized at the Bam HI site, and equal amounts of viral DNA were transfected into permissive SVG-A cells by Fugene HD (Roche Diagnostics) reagents according to the manufacturer's instructions. Viral growth at the indicated time points was monitored by indirect immunofluorescence of VP1. For quantification of viral particle release, the culture supernatant was collected at each indicated time point and ultracentrifuged in a Beckman TLA-100.3 rotor at 80,000 rpm for 60 min at 4°C. The pellet fraction and whole cell lysates (WCL) were analyzed simultaneously by immunoblotting. Results were confirmed by at least three independent experiments.

### Confocal Microscopy

Cells were fixed with 3% paraformaldehyde (PFA) in phosphate-buffered saline (PBS), permeabilized with 0.5% Triton X-100 in PBS, and incubated at room temperature with 1% BSA in PBS. The cells were then incubated with primary antibodies, followed by Alexa 488– or Alexa 594–labeled goat antibodies to rabbit IgG or with Alexa 488– or Alexa 594–labeled goat antibodies to mouse IgG (Molecular Probes). The cells were observed with a confocal laser-scanning microscope (Olympus, Tokyo, Japan).

For analysis of colocalization between GST-EGFP proteins and DsRed-ER, the 293T cells transfected with pGST-EGFP-Agno or mutant plasmid and pDsRed-ER were fixed with 3% PFA in PBS at 48 h post-transfection. The cells were then observed with a confocal laser-scanning microscope. The fluorescence intensity along a white straight line of the captured images was measured by using FV10-ASW ver.1.6 imaging software (Olympus). Cell surface immunofluorescent staining was previously described [49]. Briefly, cells were incubated in DMEM with 10% fetal calf serum and an appropriate primary antibody for 1 h at 4°C. After washing three times with cold PBS, the cells were fixed with 3% PFA and then stained with Alexa488-conjugated anti-mouse IgG antibody.

### Analysis of the Association of Proteins with Membranes

Microsome fractions were prepared essentially as described previously [30]. Briefly, 293AG cells in five 10 cm-diameter dishes were homogenized with a Dounce homogenizer in 5 ml of homogenizing buffer (0.25 M sucrose and 20 mM Hepes-NaOH) supplemented with Complete protease inhibitor cocktail (Roche). The homogenate was centrifuged at 1,000×g for 10 min at 4°C. The supernatant was centrifuged at 2,000×g for 30 min at 4°C. The supernatant was then ultracentrifuged in a Beckman TLA-100.3 rotor at 85,000 rpm for 30 min at 4°C. The pellet (microsomes) and the supernatant (cytosol) fractions were mixed with an equal volume of 2 × SDS-PAGE sample buffer and analyzed by immunoblotting.

To analyze the association of proteins with membranes, the pellet fraction (microsomes) were further incubated with 100 µl of 1 M KCl, 0.2 M sodium carbonate (pH 11), or 2 M Urea in the buffer for 1 h on ice and then recovered by ultracentrifuging in a Beckman TLA-100.3 rotor at 85,000 rpm for 30 min at 4°C. The supernatant and pellet fractions were then analyzed by immunoblotting. Triton X-114 phase separation was performed as described previously [30]. Briefly, the microsomes thus prepared were incubated in the Tx114-lysis buffer [1% Triton X-114, 10 mM Tris-HCl (pH 7.4), 150 mM NaCl, and 1 mM EDTA] with Complete protease inhibitor cocktail for 60 min at 4°C. The lysate was centrifuged at 10,000×g for 15 min at 4°C. The supernatant was incubated at 37°C for 3 min and then centrifuged at 10,000×g for 1 min at 4°C. The upper (aqueous) phase, the lower (detergent) phase and the pellet were analyzed by immunoblotting.

### Iodixanol Density Gradient Analysis

In order to analyze the membranous fraction from JCI cells, cells in a 10 cm-diameter dish were suspended in 1 ml of the homogenization buffer A [0.25 M sucrose and 60 mM HEPES-NaOH (pH 7.4)] containing protease inhibitor (Complete Mini, EDTA-free, Roche Diagnostics). The cells were homogenized using a tight-fitting Dounce homogenizer (5 strokes), and the homogenates were centrifuged at 400×g for 3 min to remove the nuclei and cell debris. The supernatant was treated with a solution containing 20 µg/ml DNase I, 7.5 mM manganese chloride, and 0.5 mM DTT for 30 min at room temperature, and centrifuged at 13,000×g for 15 min at 4°C. The pellets were resuspended with 2 ml of the homogenization buffer B [0.25 M sucrose, 1 mM EDTA, and 10 mM HEPES-NaOH (pH 7.4)], and immediately used for fractionation. Iodixanol (OptiPrep, Nycomed) density gradient analysis was performed according to the manufacturer's instructions. A working solution of 50% iodixanol [5∶1 v/v mixture of OptiPrep and 0.25 M sucrose, 6 mM EDTA, 60 mM HEPES–NaOH (pH 7.4)] was diluted with the homogenization buffer to prepare a 25% solution. Iodixanol continuous gradients were formed with 5.5 ml each of 0% (the homogenization buffer containing the protease inhibitor) and 25% iodixanol solutions in 13-ml open-top centrifuge tubes. Two ml of the cell lysates was overlaid on the top of the gradients, and then ultracentrifuged in a Hitachi P40ST rotor at 40,000 rpm for 2.5 h at 4°C. The gradients were fractionated into 24 fractions of each 500 µl from the top, and analyzed by immunoblotting.

Quantification of the intensities of obtained bands by immunoblotting was performed using the Image Gauge V3.2 software (Fuji Film, Tokyo, Japan).

### Flow Cytometry

293T cells were detached using an enzyme-free/PBS-based cell dissociation buffer (Gibco/BRL) according to the manufacturer's instruction. Aliquots of 10^6^ cells were washed in PBS/2% FBS and suspended in 100 µl of PBS/2%FBS. Cells were then incubated with 200 ng of primary antibody or control IgG (BD Pharmingen) as a negative control for 30 min at 4°C. After washing, bound antibodies were visualized by addition of phycoerythrin (PE)-conjugated anti-mouse or -rabbit Ig antibody (Beckman Coulter). After washing, cells were suspended in 250 µl of PBS/2% FBS. Cell surface fluorescence was analyzed with a Becton Dickinson FACScalibur (BD Bioscience, San Jose, California).

### Transfection, Immunoblot Analysis, and Immunoprecipitation

Cell transfection was performed with Lipofectamine 2000 (Invitrogen) for 293T cells or Fugene HD for HeLa and SVG-A cells. For immunoblot analysis, cells were harvested at the indicated time points after transfection, lysed in TNE buffer [10 mM Tris-HCl (pH 7.5), 150 mM NaCl, 5 mM EDTA, 10% glycerol, 1% Triton X-100, and 0.5 mM phenylmethylsulfonyl fluoride (PMSF)], and mixed with Complete protease inhibitor cocktail. The cell lysates were fractionated by SDS-PAGE, and the separated proteins were transferred to a polyvinylidene difluoride filter (Millipore). The filter was incubated with primary antibodies, and immune complexes were then detected with horseradish peroxidase–conjugated secondary antibodies and ECL reagents (GE Healthcare). The Flag epitope was detected directly with horseradish peroxidase–conjugated primary antibodies.

For detection of the homo-interaction of agnoproteins, 293T cells transfected with Myc-tagged agnoprotein/Flag-tagged agnoprotein or Myc-tagged agnoprotein alone were incubated for 72 h and then lysed in TNE buffer and subjected to immunoprecipitation.

Immunoprecipitation was performed by incubation of cell lysates at 4°C first for 4 h with antibody-coupled protein G–Sepharose FF beads (GE Healthcare). After washing with cell lysis buffer, the bead-bound proteins were subjected to immunoblot analysis.

### Preparation of an ER-Nuclear Fraction

Subcellular fractionation was performed by a procedure that allows separation of nuclei and ER from other membrane fractions. Cells were suspended in ice-cold sucrose buffer I [0.32 M sucrose, 3 mM CaCl_2_, 2 mM magnesium acetate, 0.1 mM EDTA, 10 mM Tris-HCl (pH 8.0), 1 mM dithiothreitol (DTT), and 0.5% Nonidet P-40] and then mixed with sucrose buffer II [1.8 M sucrose, 5 mM magnesium acetate, 0.1 mM EDTA, 10 mM Tris-HCl (pH 8.0), and 1 mM DTT]. The resulting mixture was gently overlaid on 4.4 ml of sucrose buffer II in a 13-ml open-top centrifuge tube to form a discontinuous sucrose gradient. The gradient was centrifuged at 30,000×g for 45 min at 4°C, and the resulting pellet was washed with PBS containing 0.5% Triton X-100. The pellet was then lysed in nuclear lysis buffer [25 mM Tris-HCl (pH 7.4), 300 mM NaCl, 0.5% Nonidet P-40, and 0.5% sodium deoxycholate] and rotated for 1 h at 4°C. The lysate was centrifuged at 20,000×g for 30 min at 4°C, and the resulting supernatant was subjected to immunoblot analysis.

### Chemical Cross-Linking

Crosslinking of agnoprotein in SVG-A cells infected with JCV was performed as described previously with minor modifications [50]. SVG-A cells infected with JCV for 1 week were detached using an enzyme-free/PBS-based cell dissociation buffer (Gibco/BRL) according to the manufacturer's instruction. Aliquots of 10^6^ cells were washed in DPBS (+) and suspended in 100 µl of PBS/2% FBS. The cells were suspend in 100 µl of DPBS (+) and then reacted with 0.5, 1, 2 or 5 mM disuccinimidyl suberate (DSS, Pierce) or DMSO for 2 h at 4°C. The cells were quenched with 50 mM Tris (pH 7.5) for 30 min at 4°C and then lysed in 1% Triton X-100 in PBS at 4°C and centrifuged at 15,000×g for 15 min at 4°C. Supernatants were subjected to SDS-PAGE and immunoblot analysis using anti-agnoprotein antibody.

### Intermolecular FRET Signal Correction and Normalization

The method of analyzing the oligomerization of agnoprotein was adapted from a previously published method [51]. SVG-A cells plated on collagen-coated 35-mm diameter glass base dishes (Asahi Techno Glass, Tokyo, Japan) were transfected with pCXSN-YFP-Agno/pCXSN-CFP-Agno or pCXSN-YFP/pCXSN-CFP. The cells were then placed in a chamber box on a microscope, in which the temperature was maintained at 37°C, and were imaged with an IX71 inverted microscope (Olympus), as described previously [52]. The analysis of the cell image data, FRET signal correction and normalization was conducted as reported previously [51].

### Measurement of Lipid Packing of Plasma Membrane

The lipid packing of plasma membrane can be studied by inserting the fluorescence probe, MC540 (Sigma) into cell membranes and assessing the degree of insertion by fluorescence intensity measurement using flow cytometry [34] [35]. HeLa cells were transfected with pCXSN-Agno or pCXSN vector, and were incubated for 72 h. The cells were then detached by using an enzyme-free/PBS-based cell dissociation buffer. The cells were suspended in 10 µg/ml MC540/DPBS (+). After 10 min incubation at 37°C, cells were washed in DPBS (+). After washing, cells were suspended in 250 µl of DPBS (+). Cell surface fluorescence was analyzed with a Becton Dickinson FACScalibur. FACScalibur was used at 488 nm excitation and 575 nm emission wavelengths.

### Hygromycin B (HygB) Permeability Assay

Permeability of the plasma membranes of agnoprotein-transfected cells to HygB (Clontech) was determined as described previously [37]. Briefly, HeLa cells or SVG-A cells were plated on a 35-mm dishes and transfected with pCFPNLS-Agno, pCFPNLS-N46, pCFPNLS-RK8AA, or pCFPNLS. At 72 h post-transfection, the cells were pretreated with HygB (400 µg/ml) for 15 min, and then 50 µCi of [^35^S] Met-Cys were added to the culture medium. The cells were then incubated at 37°C for 2 h in the presence or absence of HygB. The cell extracts were lysed in 500 µl of RIPA buffer mixed with protease inhibitor and subjected to immunoprecipitation with goat anti-GFP antibody-coupled protein G-Sepharose FF beads. The bead-bound proteins were analyzed by SDS-PAGE and autoradiography.

### Determination of Ca^2+^ Influx into Cells

The method of analyzing the rate of Ca^2+^ influx was adapted from a previously published method [38]. HeLa cells plated on a collagen-coated 35-mm diameter glass base dish were transfected with pERedNLS-Agno/pERedNLS and a FRET-based fluorescence indicator for Ca^2+^, YC3.60 (kindly provided by Dr. A. Miyawaki). After 96 h, cells were washed once in the recording medium [10 mM Hepes-NaOH (pH 7.4), 140 mM NaCl, 5 mM KCl, 1 mM MgCl_2_, and 0.55 mM Glucose]. The cells were incubated with the calcium chelater dimethyl-BAPTA-AM (Molecular Probes) for 30 min at 37°C to prolong the linear phase of unidirectional Ca^2+^ uptake. The cells were then placed in a chamber box on a microscope, in which the temperature was maintained at 37°C, and were imaged with an IX71 inverted microscope, as described previously [52]. Fluorescent images of CFP and FRET were recorded every 10 sec. At 100 sec, 5 mM CaCl_2_ was added to the cells. MetaMorph software (Universal Image) was used for control of the CCD camera and the filter wheels, and also for the analysis of the cell image data.

### Bacterial Expression Plasmids, E. coli Membrane Permeability, and Viability

Assays for E. coli membrane permeability and viability have been previously described [41]. The entire coding sequences of VP1 (pET15b-VP1) [53] and agnoprotein (pET15b-His-Agno) were amplified by PCR and cloned into the pET15b expression vector (Novagen, Madison, WI). The integrity of vectors were verified by sequencing and transformed into the E. coli BL21 strain (DE3: pLysS) for protein expression (Novagen). For permeability assays, cultures were grown in the presence of 100 µg/ml ampicillin at 37°C to an optical density (OD) at 590 nm of ∼1.0, and protein expression was induced with 1 mM isopropyl-β-D-thiogalactopyranoside (IPTG) for 10 min prior to the addition of rifampicin (150 µg/ml) to inhibit transcription of endogenous genes by the E. coli RNA polymerase. Samples were incubated for an additional 30 min followed by a 10-min pulse with 50 µCi [35S] Met-Cys in the absence or presence of 250 µg/ml hygromycin B. Bacterial cell lysis was monitored by following the OD at 590 nm after protein expression in cultures at an OD at 590 nm of ∼0.20 was induced by the addition of 1 mM IPTG.

### Electron Microscopy

Electron microscopy of JCV-infected cells was performed as follows: Cells were fixed in 2.5% glutaraldehyde (TAAB, Aldermaston, UK) in 0.1 M phosphate buffer (PB, pH 7.4) for 48 h at 4°C. Thereafter, cells were washed in the same buffer containing 7% sucrose, post-fixed in 1% osmium tetroxide (Merck, Darmstadt, Germany) in 0.1 M PB for 1.5 h at room temperature, dehydrated in graded acetones, and embedded in Epon (TAAB). Ultrathin sections (0.1 µm in thickness) were stained with 1.5% uranyl acetate for 20 min and 0.2% lead citrate for 15 min, and were examined under an H-7100 electron microscope (Hitachi, Tokyo, Japan).

### Statistical Analysis

All data were expressed as mean ± S.D. Student's *t*-test was used to analyze differences between two groups. A value of *p<0.05* was considered as statistically significant.

## Supporting Information

Figure S1 The subcellular localization of VP1 was not affected by deletion of agnoprotein. SVG-A cells transfected with wtJCV genome or ΔAgnoJCV genome were fixed 4 days post-transfection and immunostained using anti-VP1 antibody followed by Alexa 488-labeled goat anti-rabbit IgG. The cells were then immunostained with Alexa 594-labeled anti-agnoprotein antibody. Localization of VP1 in the cells was analyzed by confocal microscopy. Scale bars, 20 µm.
(4.26 MB EPS)

Figure S2 The amount of agnoprotein expression resulting from expression vector transfection is similar to that observed after infection or transfection with the JCV genome. (A) Cell lysates from SVG-A cells, SVG-A cells infected with JCV, and SVG-A cells transfected with pCXSN-Agno were analyzed by immunoblotting with an anti-agnoprotein antibody, and actin was used as an internal control. The level of agnoprotein expression in cells transfected with a plasmid encoding agnoprotein was similar to that in cells infected with JCV. (B) Cell lysates from SVG-A cells transfected with wtJCV at the indicated days post-transfection and cells transfected with pERedNLS (Mock), pERedNLS-Agno, and pCFPNLS-Agno were analyzed by immunoblotting with an anti-agnoprotein antibody, and actin was used as an internal control. The level of agnoprotein expression in cells transfected with a plasmid encoding agnoprotein was similar to that in cells transfected with the JCV genome (wtJCV). The intensity of obtained bands in (A) and (B) was quantified using Image Gauge V3.2 software (Fujifilm, Tokyo, Japan).
(0.79 MB EPS)

Figure S3 Schematic representation of the wild-type and mutant agnoprotein constructs used in Figure 5. The N-terminal region of agnoprotein is characterized by the presence of positively-charged residues. The schematic represents GST-EGFP fusion constructs of wild-type (WT) agnoprotein and various mutants. The green boxes indicate the basic amino acid clusters, which could be important for determining the orientation to the membrane. A gray box indicates a hydrophobic amino acid stretch.
(0.39 MB EPS)

Figure S4 Agnoprotein enhances the membrane permeability for HygB. (A) SVG-AG cells, which are agnoprotein-inducible with doxycycline (DOX) treatment, were established using the Retro-X™ Tet-On Advanced Inducible Expression System (Clontech). SVG-AG cells were incubated with or without 1 µg/ml DOX for 72 h. Nascent protein synthesis in these cells with HygB at the indicated concentrations was quantified using Click-iT AHA Alexa Fluor 488 Protein Synthesis Kit (Invitrogen) and FACScanto (BD Bioscience). (B) E. coli viability was monitored following the induction of VP1 and His-agnoprotein expression.
(1.26 MB EPS)

Figure S5 Comparison of wtJCV and RK8AAJCV. (A) wtJCV- or RK8AAJCV-transfected SVG-A cells were fixed at 4 days after transfection. The cells were immunostained using anti-agnoprotein antibody followed by Alexa 594-labeled goat anti-rabbit IgG and analyzed by confocal microscopy. Localization of agnoprotein in cells transfected with RK8AAJCV was similar to that in cells transfected with wtJCV. (B) wtJCV- or RK8AAJCV-transfected SVG-A cells were harvested and lysed 4 days after transfection. Immunoblot analysis was performed with antibodies to agnoprotein and actin. The level of agnoprotein expression in cells transfected with RK8AAJCV was similar to that in cells transfected with wtJCV. (C) The infectivity of virus extracted from wtJCV- or RK8AAJCV-transfected cells (cell-associated virus) was examined by the infection assay in SVG-A cells. Four days after inoculation with cell-associated virus, the cells were subjected to immunofluorescent analysis using anti-VP1 antibody. The cells were visualized with Alexa Fluor 594-conjugated goat anti-rabbit IgG. After cell nuclei were counterstained with DAPI, immunofluorescent images were visualized by confocal microscope FV1000 (Olympus). JCV VP1 was observed in cells infected by virus isolated from both wtJCV- and RK8AAJCV-transfected cells, suggesting that the generated virus is infectious and not the defective RK8AAJCV mutant.
(1.64 MB EPS)

Figure S6 Trans-expressed agnoprotein complements virus release by viroporin-deficient virus. (A) SVG-AG cells, which are agnoprotein-inducible with doxycycline (DOX) treatment, were transfected with ΔAgnoJCV genome and incubated for 72 h. The cells were then incubated with or without DOX for another 48 h. Whole cell lysates (WCL) and culture supernatants (SUP) from the cells were analyzed by immunoblotting with anti-VP1, anti-agnoprotein, and anti-actin antibodies. The amount of VP1 in the culture supernatant from cells treated with DOX was substantially increased compared to those without DOX, which is consistent with the presence of agnoprotein and suggests that trans-expressed agnoprotein complements virus release by agnoprotein-deficient JCV. (B) SVG-A cells stably expressing either N46, which possesses viroporin activity, or RK8AA, an agnoprotein mutant which lacks viroporin activity, were transfected with RK8AAJCV genome and incubated for 5 days. Whole cell lysates (WCL) and culture supernatants (SUP) from the cells were analyzed by immunoblotting with anti-VP1and anti-actin antibodies. The amount of VP1 in the culture supernatant from the cells expressing N46 was substantially increased compared to that of RK8AA or Mock cells, suggesting that trans-expressed viroporin complements virus release of viroporin-deficient JCV.
(0.61 MB EPS)

Table S1 Constructs and materials used in the experiments.
(0.07 MB PDF)

Video S1 HeLa cells transfected with pERedNLS-Agno and FRET probe (YC3.60). Cells coexpressing pERedNLS-Agno (Video S1) or pERedNLS (Video S2) and YC3.60 were incubated for 72 h. Permeability to Ca^2+^ in agnoprotein-expressing (Video S1) or mock-transfected (Video S2) cells was evaluated by the change in FRET ratio induced by the addition of 5 mM CaCl_2_ to the extracellular medium 100 sec after the start of recording. Fluorescent images of CFP and FRET were recorded every 10 sec for 15 min. Ratio images of FRET/CFP were created to represent FRET efficiency. In the IMD mode shown here, eight colors from red to blue are used to represent the FRET/CFP ratio. The upper (red) and lower (blue) limits of the ratio range are 4.5 and 2.6, respectively.
(3.58 MB MOV)

Video S2 HeLa cells transfected with pERedNLS and FRET probe (YC3.60).
(2.48 MB MOV)
