# Supplementary material for: The Human Polyoma JC Virus Agnoprotein Acts as a Viroporin
Source: PLoS Pathog. 2010 Mar 12;6(3):e1000801. doi: 10.1371/journal.ppat.1000801 (PMC2837404; doi:10.1371/journal.ppat.1000801)
Supplement: Table S1 — Constructs and materials used in the experiments. (0.07 MB PDF) [file ppat.1000801.s007.pdf]

Figure	Constructs / Materials
Figure 1AB	SVG-A cells transfected with JCV WT genome (WT) /JCV ΔAgno genome (ΔAgno)
Figure 2A	SVG-A cells inoculated with JCV
Figure 2B	IMR-32 cells inoculated with JCV
Figure 3ABC	293AG cells: agnoprotein-inducible cell lines with doxycycline treatment
Figure 3DE	SVG-A cells inoculated with JCV inoculation
Figure 3FG	pERedNLS-Agno (Agno)/pERedNLS (Mock):<br>pERedNLS contains the internal ribosome entry site (IRES) of the encephalomyocarditis virus (ECMV) between the multiple cloning sites (MCS) and the ERedNLS (DsRed-Express with nuclear localization signal (NLS) of simian virus 40 large T-antigen fused to its C-terminus). This plasmid permits both the inserted gene into the MCS and the ERedNLS gene to be translated from a single bicistronic mRNA. These plasmids-transfected cells were thus labeled by expression of DsRed-Express in the nucleus.<br>Agno is not fused with ERedNLS, but tagged Flag-epitope at its NH2-ternimus.
Figure 3H	293T cells transfected with pCXSN-Agno (Agno)/pCXSN (Mock)
Figure 4	HEK293 cells transfected with<br>Agno (WT)-GST-EGFP/C6-GST-EGFP/N46-GST-EGFP/GST-EGFP: Agno and deletion mutants are fused with GST-EGFP at its COOH-terminus.
Figure 5	293T cells transfected with pDsRed-ER and Agno<br>(WT)-GST-EGFP/C6-GST-EGFP/C18-GST-EGFP/<br>N24-GST-EGFP/N46-GST-EGFP/R4A-GST-EGFP/<br>RK8AA-GST-EGFP/KKR22AAG-GST-EGFP/GST-EGFP
Figure 6A	293T cells transfected with pCXSN-Myc-Agno/pCXSN-Flag-Agno: Agno is tagged with Myc-epitope or Flag-epitope at its NH2-terminus, respectively.
Figure 6B	SVG-A cells inoculated with JCV
Figure 6CD	SVG-A cells transfected with pCXSN-Venus-Agno/ pCXSN-CFP-Agno:<br>Agno is fused with Venus/sECFP at its NH2-terminus.
Figure 7A	HeLa cells transfected with pCXSN-Agno/pCXSN (Mock)
Figure 7BC	HeLa cells transfected with pCFPNLS-Agno/pCFPNLS (Mock): pCFPNLS contains the IRES of ECMV between the MCS and the CFPNLS (sECFP with NLS) of simian virus 40 large T-antigen fused to its C-terminus. This plasmid permits both the inserted gene into the MCS and the CFPNLS gene to be translated from a single bicistronic mRNA. These plasmids-transfected cells were thus labeled by expression of sECFP in the nucleus.<br>This plasmid was generated from pERedNLS by replacement of ERedNLS with sECFP.<br>Agno is not fused with CFPNLS, but tagged Flag-epitope at its NH2-ternimus.
Figure 7DEF	HeLa cells transfected with pERedNLS-Agno/pERedNLS (Mock)
Figure 8A	HeLa cells transfected with pCFPNLS-Agno (WT) /N46/RK8AA/pCFPNLS (Mock)
Figure 8BCD	SVG-A cells transfected with JCV WT genome (WT)/<br>JCV RK8AA genome (RK8AA)
